# Nitric oxide triggers the assembly of “type II” stress granules linked to decreased cell viability

**DOI:** 10.1038/s41419-018-1173-x

**Published:** 2018-11-13

**Authors:** Anaïs Aulas, Shawn M. Lyons, Marta M. Fay, Paul Anderson, Pavel Ivanov

**Affiliations:** 1 Division of Rheumatology, Immunology, and Allergy, Brigham and Women’s Hospital, Boston, MA 02115 USA; 2 Department of Medicine, Harvard Medical School, Boston, MA 02115 USA; 3 The Broad Institute of Harvard and M.I.T., Cambridge, MA 02142 USA

## Abstract

We show that 3-morpholinosydnonimine (SIN-1)-induced nitric oxide (NO) triggers the formation of SGs. Whereas the composition of NO-induced SGs is initially similar to sodium arsenite (SA)-induced type I (cytoprotective) SGs, the progressive loss of eIF3 over time converts them into pro-death (type II) SGs. NO-induced SG assembly requires the phosphorylation of eIF2α, but the transition to type II SGs is temporally linked to the mTOR-regulated displacement of eIF4F complexes from the m^7^ guanine cap. Whereas SA does not affect mitochondrial morphology or function, NO alters mitochondrial integrity and function, resulting in increased ROS production, decreased cytoplasmic ATP, and plasma membrane permeabilization, all of which are supported by type II SG assembly. Thus, cellular energy balance is linked to the composition and function of NO-induced SGs in ways that determine whether cells live or die.

## Introduction

A conserved feature of the integrated stress response is a global translation inhibition leading to stress granule (SG) formation^1^. SGs are membrane-less cytoplasmic foci containing translationally stalled mRNA, RNA binding proteins, and signaling molecules. Many SG components are in dynamic equilibrium with polysomes, allowing cells to rapidly modulate protein translation in response to changing environmental conditions. Recently, we reported that different stress conditions induce the assembly of compositionally distinct SG sub-types suggesting a difference in function that was not yet investigated^2^. While NO is known to induce general translation arrest^3^, the mechanism of translation repression and its ability to trigger SG assembly has not been studied. Here, we challenged cells with 3-morpholinosydnonimine (SIN-1), a commonly used NO donor^4^. We show that NO induces the assembly of non-canonical, type-II, SGs that lack eIF3. NO-induced SGs are less dynamic than canonical type-I SGs and their assembly correlates with the disruption of mitochondria, ATP depletion, and increased cell permeability.

Previously, we reported a correlation between the assembly of eIF3-deficient SGs and increased cell death; however, those studies were descriptive and did not assess the molecular mechanism involved^5,6^. Our current observations coupled with our previous report have allowed us to define two SG sub-types: type-I SGs such as those induced by SA include eIF3 and promote cell survival, whereas type II SGs induced by selenite or NO exclude eIF3 and are linked to enhanced cell death. The formation of both type-I and type-II SGs depend upon inhibition of global translation; however, it is worth noting that translation arrest is not sufficient for SG formation. Our findings strongly support a model in which type-I SGs are converted to type-II SGs when mitochondrial damage leads to increased ROS (reactive oxygen species) and decreased ATP, metabolic changes that promote the egress of eIF3. As such, the assembly of type-II SGs is a marker of energy depletion and cell death.

## Materials and methods

### Cell culture and treatment

U2OS (previously used by our laboratory in the ref.^7^), HAP1 (previously used by our laboratory in the ref.^2^) and MEFs (previously used by our laboratory in the ref.^8^) cells were maintained at 37 °C in a CO_2_ incubator in Dulbecco’s modified Eagle’s medium high glucose (25 mM, Gibco, Waltham, MA, USA) supplemented with 10% fetal bovine serum (Sigma, Saint Louis, MO, USA), 20 mM HEPES (Gibco, Waltham, MA, USA) and 1% penicillin/streptomycin (Gibco, Waltham, MA, USA). For SG induction, cells were grown to ∼70% confluency and then treated with 3-Morpholinosydninimine (SIN-1, Santa Cruz, Dallas, TX, USA), S-Nitroso-*N*-acetylpenicillamine (SNAP, Santa Cruz, Dallas, TX, USA), sodium arsenite (SA) (100 µM, Sigma, Saint Louis, MO, USA) or menadione (Sigma, Saint Louis, MO, USA) at indicated concentrations. Puromycin (20 µg/ml) and cycloheximide (50 µg/ml) treatment was performed 30 min before collecting the coverslips or as described in the text. ISRIB (Sigma, Saint Louis, MO, USA) was used at 2 nM , PTIO (EMD Millipore, Darmstadt, Germany) was used at 5 mM, and NAC (Sigma, Saint Louis, MO, USA) was used at 5 mM.

### In vitro labeling

For ribopuromycylation, cells were pulsed with puromycin (5 µg/ml) for 5 min before lysis. Determination of ROS production was performed using CellRox deep red reagent (Life Technologies, Waltham, MA, USA) at 2.5 µM concentration 30 min before fixation. Mitochondria were labeled using MitoTracker deep red (Invitrogen, Carlsbad, CA, USA) at 1 µM for 15 min before fixation.

### Western blotting

Following drug treatment, cells were washed with phosphate buffered saline (PBS) and sonicated in lysis buffer (50 mM Hepes [pH 7.6], 150 mM NaCl, 0.5% NP-40, 5% Glycerol) with HALT phosphatase and protease inhibitors (Thermo Scientific, Waltham, MA, USA). Laemmli’s sample buffer supplemented with 100 mM dithiothreitol (DTT) was added to samples to 1× final concentration. Samples were boiled and loaded into a 4–20% Tris–Glycine gel (BioRad, Hercules, CA, USA), transferred to nitrocellulose membrane. Antibody detection was performed using SuperSignal West Pico Chemiluminescent Substrate (Thermo Scientific, Waltham, MA, USA).

### Cap binding assay

Assembly of eIF4E-containing complexes from untreated U2OS cell lysates or lysates treated with H_2_O_2_ (1 mM, 1 h) or SA (100 μM, 1 h) was performed as described in the ref. ^9^.

### ^35^S-Met labeling

For mitochondrial translation assessment, media was exchanged to DMEM lacking leucine or methionine for 30 min before addition of emetine (100 µg/ml) for 5 min. Then Expre^35^S^35^S labeling mix (Perkin Elmer, Waltham, MA, USA) was added to a final concentration of 200 µCi/ml to the leucine- or methionine-free media containing emetine (100 µg/ml) for 60 min. Cells were collected, and lysates (40–50 μg) were run on a 4-20% SDS gradient gel. Gels were stained with Coomassie blue, incubated with EnH^3^ance (National Diagnostics, Atlanta, GA USA) following manufacturer instructions and dried before to exposed to radiographic films.

### Polysome profiling

Cells were treated with 100 μg/ml cycloheximide for 10 min, washed with HBSS, and harvested into lysis buffer (10 mM Tris [pH 7.4], 150 mM NaCl, 5 mM MgCl_2_, 1 mM DTT, 100 μg/ml cycloheximide, 1% Triton-X100 in DEPC-treated water) supplemented with RNasin Plus inhibitor (Promega, Madison, WI USA) and HALT phosphatase and protease inhibitors (Thermo Scientific, Waltham, MA, USA). Lysates were rotated at 4 °C for 10 min, and centrifuged for 10 min at 10,000×*g*. Supernatants were loaded onto 10–50 % sucrose gradients made in gradient buffer (150 mM NaCl, 20 mM Tris [pH 7.4], 5 mM MgCl_2_, 1 mM DTT, 100 µM cycloheximide, 0.25% NP40, RNasin) and centrifuged in a Beckman SW55Tirotor for 1.5 h, 45,000x*g* at 4 °C. Samples were eluted using a Brandel bottom-piercing apparatus attached to a syringe pump. An ISCO UV monitor was used to measure the eluate at OD 254.

### Semi-quantitative qRT-PCR

Polysomal fractions were collected. RNA was extracted using Trizol LS and reverse transcribed using the QuantiTech Reverse Transcription kit (Qiagen, Hilden, Germany). qRT-PCR was performed using the following primer sets:

Actin: 5′-CCTGGATAGCAACGTACATGG-3′; 5′-ACCTTCTACAATGAGCTGCG-3′;

ATP5O: 5′-TCCTGAAGGAACCCAAAGTG-3′; 5′-ATCGACCATTTTCAGCAAGC-3′;

TFAM: 5′-CCGAGGTGGTTTTCATCTGT-3′; 5′-TCCGCCCTATAAGCATCTTG-3′

### Immunofluorescence

Cells were grown on coverslips, subjected to the indicated treatments, washed with PBS, fixed with 4% paraformaldehyde for 15 min at room temperature. Cells were permeabilized using −20 °C methanol for 5 min. Coverslips were blocked with 5% normal horse serum in PBS for at least 30 min. Primary antibodies (Table 1) were diluted in blocking buffer and incubated overnight at 4 °C or 1 h at room temperature. Coverslips were washed three times for 5 min between primary and secondary antibody incubations. Subsequently, secondary antibodies (Table 1) were added along with Hoechst Dye for 1 h at room temperature. Cells were washed extensively and mounted with Vinol.

**Table 1:** Antibodies list

Antibodies	Suplier	Cat number	WB dilution	IF dilution
4EBP1 total	Cell Signaling	9644 S	1/3000	
4EBP1 non-phosphorylated form	Cell Signaling	4923 S	1/1000	
Actin	ProteinTech Group	66009-1-G	1/10,000	
ATP5D	ProteinTech Group	14893-1-AP	1/1000	
ATP5O	Bethyl	A305-418A-T	1/1000	
ATP8	ProteinTech Group	26723-1-AP	1/1000	
Caspase 3	Cell Signaling	9662 S	1/200	
COXIV	ProteinTech Group	11242-1-AP	1/1000	
CYTB	ProteinTech Group	55090-1-AP	1/1000	
eIF2α	Cell Signaling	2103 S	1/1000	
eIF3B	Santa Cruz Bio	sc-16377		1/250
eIF2α phosphorylated form	Abcam	ab131505	1/1000	
eIF3J	ProteinTech Group	10439-1-AP		1/500
eIF3G	ProteinTech Group	11165-1-AP		1/500
eIF3E	Bethyl	A302-985-A-M	1/1000	
eIF3D	Bethyl	A301-759-A-M	1/500	
eIF3D	ProteinTech Group	10219-1-AP		1/500
eIF4A	Santa Cruz Bio	SC-377315	1/1000	
eIF4E	Santa Cruz Bio	SC-9976	1/1000	
eIF4G	Santa Cruz Bio	SC-11373		1/200
FXR1	Santa Cruz Bio	SC-10554		1/200
G3BP1	Santa Cruz Bio	SC-365338		1/200
HuR	Santa Cruz Bio	SC-5261		1/100
ND1	ProteinTech Group	19703-1-AP	1/1000	
Mitochondria-specific	Immunovision	HMS-0300		1/1000
OMA-1	ProteinTech Group	17116-1-AP	1/1000	
OPA-1	BD Biosciences	612606	1/1000	
PABP	Santa Cruz Bio	SC-32318		1/100
PARP1	Bethyl	A301-376-A-M	1/500	
Puromycin-specific	Millipore	MABE343	1/1000	
RACK1	Santa Cruz Bio	SC-17754		1/150
TFAM	ProteinTech Group	23996-1-AP	1/1000	
TIA-1	Santa Cruz Bio	sc-1751		1/250
TOM40	ProteinTech Group	18409-1-AP	1/1000	

### Fluorescence in situ hybridization (FISH)

Fluorescence in situ hybridization was performed as described in the ref. ^10^. Briefly, cells were fixed with 4% paraformaldehyde for 15 min then permeabilized with −20 °C methanol for 5 min. Cells were incubated overnight in 70% ethanol at 4 °C. The following day, cells were washed twice with 2× saline-sodium citrate (SSC, Ambion, Waltham, MA USA), blocked in hybridization buffer (Sigma, Saint Louis, MO, USA) for 30 min. Hybridization was performed using 2 ng/µl of biotinylated oligo-dT_40×_ probe diluted in hybridization buffer at 37 °C. After extensive washes with 2 × SSC at 37 °C the probe was revealed using a fluorophore-conjugated streptavidin antibodies (Jackson Immunoresearch Laboratories, West Grove, PA, USA), followed by immunostaining as described above.

### Fluorescence recovery after photobleaching

U2OS^ΔG3BP1^ reconstituted with GFP-tagged G3BP1 were plated 24 h prior stress. Cells were stressed as indicated and transferred to the live imaging chamber (37 °C, 5% CO_2_, humidified) 30 min before starting the experiment. Three frames were collected before bleaching and 20 after, at 5 s intervals. The photobleaching beam was positioned directly over selected SG, and laser power were turn to 100% of the power to perform bleaching.

### BrdU incorporation

Cells were plated on coverslips the day before the experiment. The experiment was performed using the ApoBrdU DNA Fragmentation Assay kit from BioVision (San Franscico, CA, USA) according to the manufacturer instructions.

### Calcein AM

Cells were plated on coverslips the day before the experiment. Cells were incubated with 5 µM Calcein Blue AM (eBioscience, San Diego, CA, USA) for the last 5 min and extensively washed. Images were taken immediately after mounting coverslips in 1× PBS with 20% glycerol.

### Microscopy

Wide-field fluorescence microscopy was performed using an Eclipse E800 microscope (Nikon, Minato, Tokyo, Japan) equipped with epifluorescence optics and a digital camera (Spot Pursuit USB). Image acquisition was done with a 40× objective (PlanApo; Nikon, Minato, Tokyo, Japan).

### Electron microscopy

Electron microscopy was performed by the electron microcopy core facility at Harvard Medical School. Briefly, cells were fixed for at least overnight at 4 °C in fixative (2.5% Glutaraldehyde, 1.25 % Paraformaldehyde and 0.03% picric acid in 0.1 M sodium cacodylate buffer [pH 7.4]), washed in 0.1 M cacodylate buffer and post-fixed with 1% Osmium tetroxide (OsO_4_)/1.5 % potassium ferrocyanide (KFeCN_6_) for 1 h, washed twice with water, once with 1× Maleate buffer (MB) and incubated in 1 % uranyl acetate in MB for 1 h, followed by two washes in water and subsequent dehydration in the selected grades of alcohol (10 min each; 50, 70, 90 %, 2 × 10 min 100 %). The samples were then put in propyleneoxide for 1 h and infiltrated overnight in a 1:1 mixture of propyleneoxide and TAAB Epon (Marivac Canada Inc. St. Laurent, Canada). The following day the samples were embedded in TAAB Epon and polymerized at 60 °C for 48 h.

Ultrathin sections (about 60 nm) were cut on a Reichert Ultracut-S microtome, picked up on to copper grids stained with lead citrate and examined in a JEOL 1200EX Transmission electron microscope or a TecnaiG² Spirit BioTWIN and images were recorded with an AMT 2k CCD camera.

### Data analysis

SGs were visualized by IF with indicated markers. For quantifications, three fields were taken from selected samples, with a minimum of three replicated experiments. Cells were considered SG-positive if they have at least two cytoplasmic foci. Counting was assessed with ImageJ utilizing the cell counter and plotted as percentage of the total number of cells. Data were compared via two-tailed Student *t*-test.

### ATP measurement

ATP measurement was done using CellTiter-Glo Luminescent Cell Viability Assay (Promega Madison, WI USA) following manufacturer instructions. Measuarements are made using the GloMax explorer plate reader (Promega Madison, WI USA).

### Cell death measurement

At the indicated times, cells were collected by trypsinization, and death was assessed using trypan blue exclusion cell counting. Cell permeabilization was plotted as a percentage of trypan positive cells over the total number of cells.

## Results

### NO-induced ROS triggers SG assembly

**Fig. 1:**
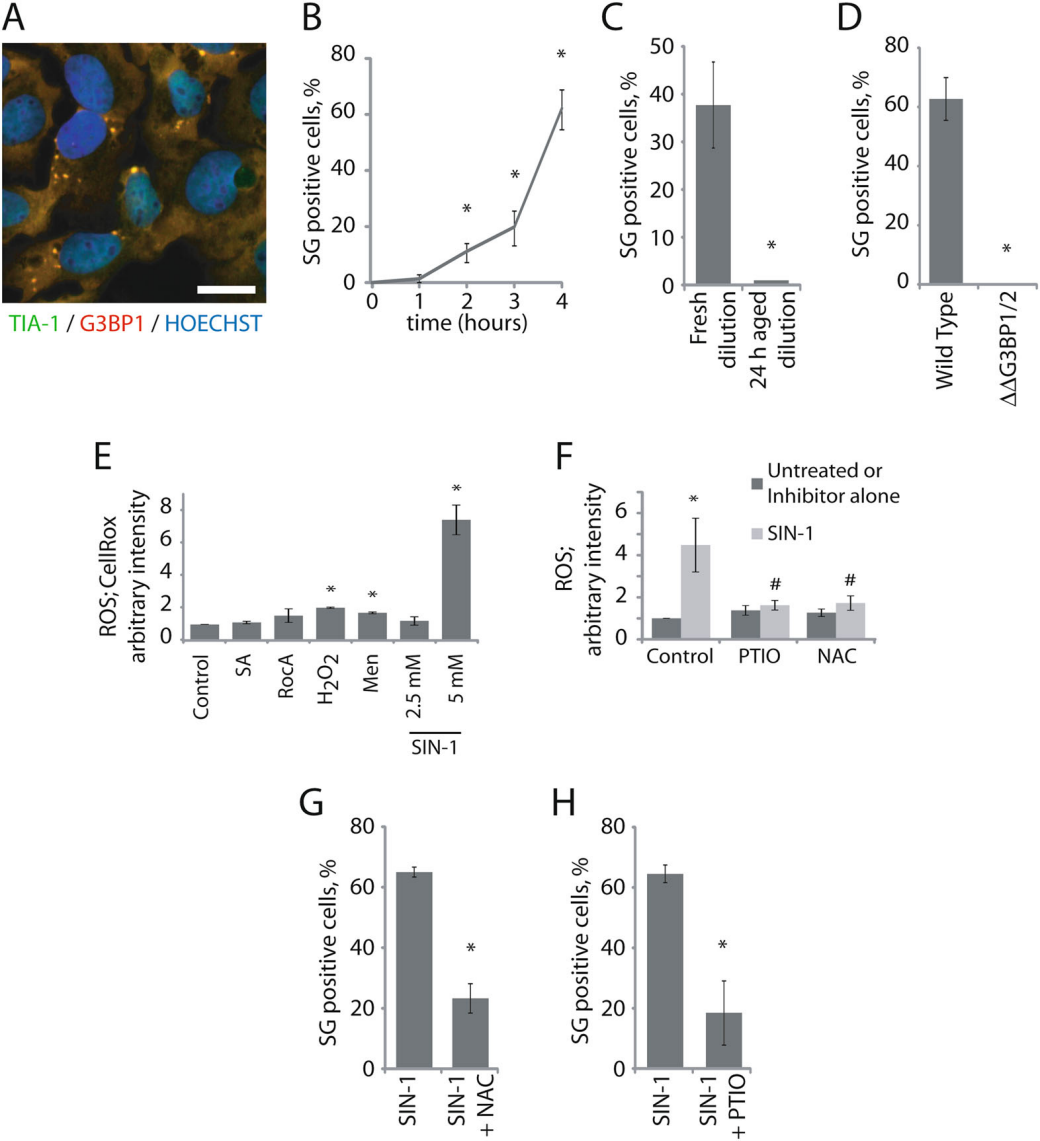
NO induces SG formation due to ROS generation. **a** Representative images of cells exposed to 5 mM SIN-1 for 4 h. Scale bar is 10 µm. **b** Cells were treated with 5 mM SIN-1 and collected every hour for 4 h and assessed by immunofluorescence for HuR and TIA-1 to identify SGs. **c** U2OS cells were treated with freshly diluted SIN-1 or 24 h old “aged” dilution for 4 h. **d** Wild type or U2OS^ΔΔG3BP1/2^ (ΔΔG3BP1/2) U2OS cells were treated with 5 mM SIN-1 for 4 h. **e**, **f** 30 min before collection, U2OS were incubated with 2.5 µM CellRox for the remaining time. Images are taken directly after fixation. CellRox intensity is expressed relative to the unstressed control intensity. **e** Cells were treated 100 µM SA (1 h), 2 µM RocA (2 h), 1 mM H_2_O_2_ (2 h), 100 μM menadiaone (Men) (1 h), SIN-1 for 4 h at the indicated concentration, or left unstressed. **f** Cells were treated with 5 mM SIN-1 for 4 h. PTIO (5 mM) or NAC (5 mM) are added to the media during the 4 h stress as indicated in the figure. **g**, **h** SG positive cells were quantified using G3BP1 and TIA-1 markers (coverslips from Fig. 1f were collected). #- comparison of cells treated with PTIO or NAC in the presence of SIN-1 versus SIN-1 only (control). Experiments are represented as mean ± SEM, *p < 0.05, *n* ≥ 3 and pictures are representative of ≥3 independent experiments

SIN-1 induces the formation of SGs in U2OS osteosarcoma cells as analyzed by colocalization of G3BP1 and TIA-1, two canonical SG markers (Fig. 1a). Unlike data previously reported for SA^7^, NO induced SGs display delayed kinetics in which SG assembly progressively increases over 4 h (Fig. 1b). Oxidative metabolism of SIN-1 results in the production of NO and accumulation of the SIN-1-derived metabolite SIN-1C^4^. Therefore, SIN-1-induced SGs could be due to accumulation of SIN-1C rather than NO. Because NO is highly labile, SIN-1 must be properly stored in a humidity-free and light-free container and solutions freshly made. Consequently, “aged” solutions contain SIN-1C, but little or no NO. The finding that “aged” solutions of SIN-1 do not induce SG assembly implicates NO in this process (Fig. 1c). Further evidence that NO is the active SG-inducing entity comes from the finding that S-Nitroso-*N*-acetylpenicillamine (SNAP), another compound that produces NO, also induces SGs (Figure S1, dark bar, and data not shown). Lastly, U2OS^ΔΔG3BP1/2^cells that do not assemble SGs in response to most stresses^7^ do not assemble SGs in response to NO (Fig. 1d).

As previously reported^11^, NO is a potent inducer of ROS (Fig. 1e). The NO-specific scavenger P2-Phenyl-4,4,5,5-tetramethylimidazoline-1-oxyl 3-oxide (PTIO)^12^ and the ROS scavenger N-acetyl-cysteine (NAC)^13^ reduce SIN-1-induced ROS production to basal levels (Fig. 1f) and strongly reduce SIN-1-induced SG assembly (Fig. 1g, h), identifying ROS as an intermediate in NO-induced SG formation. Taken together, our data reveal that SIN-1 sequentially induces the production of NO and ROS to trigger SG assembly.

### NO induces phosphorylation of eIF2α and displacement of the eIF4F complex from cap structures to inhibit translation and induce SG assembly

**Fig. 2:**
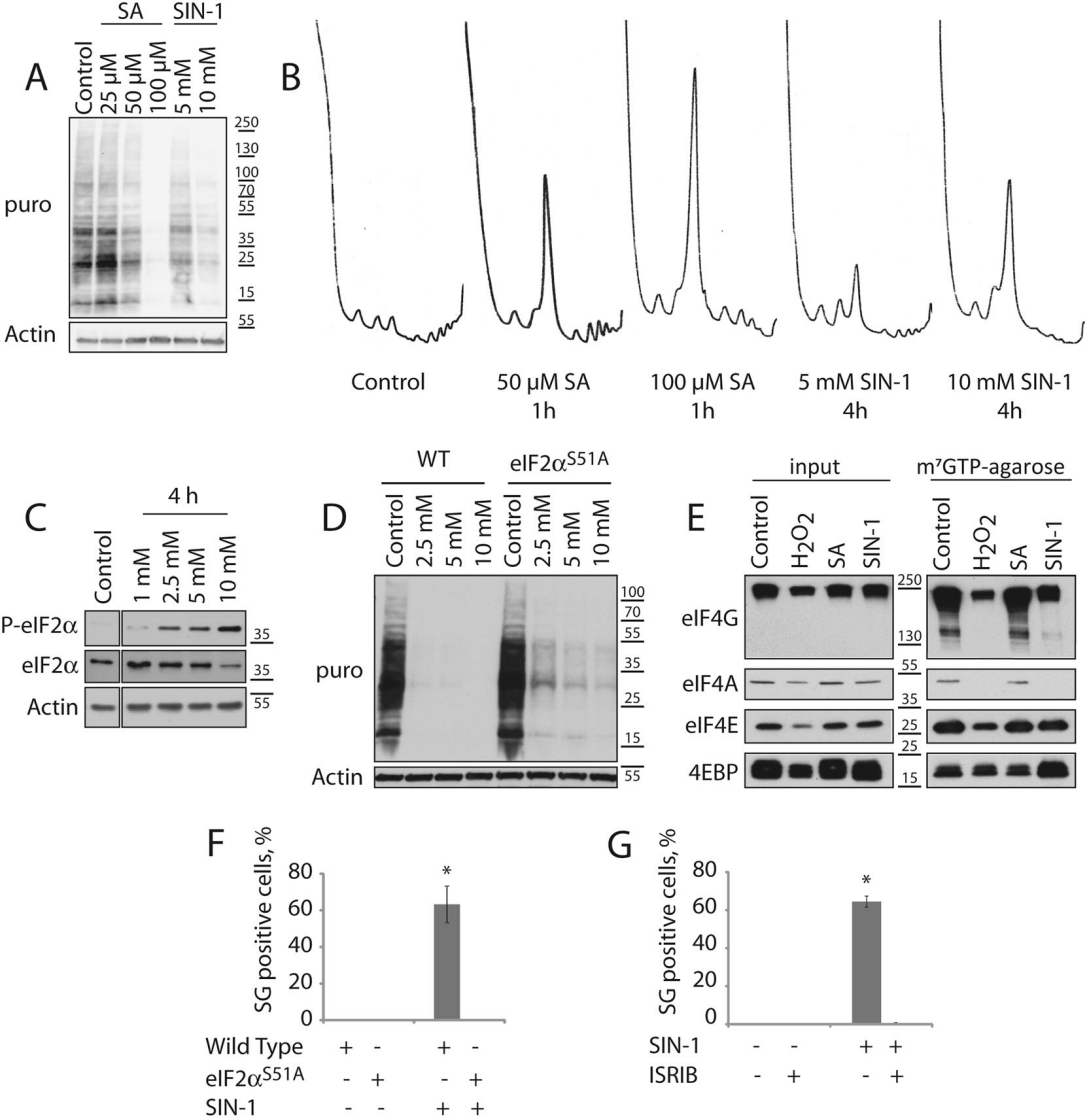
NO induces translation inhibition through phosphorylation of eIF2α and eIF4F complex displacement. **a**, **b** U2OS were treated with SA (1 h) or SIN-1 (4 h) at indicated concentrations or left untreated. **a** Cells were pulsed with puromycin (20 µg/ml) before lysis, and analyzed by western blot with a puromycin-specific antibody to assess the global translation levels and an antibody against actin as a loading control. **b** Representative polysome profiles. **c** After 4 h of SIN-1 treatment at indicated concentrations, samples were assessed for eIF2α phosphorylation, total eIF2α and actin by western blot. **d** Wild type or eIF2α^S51A^ HAP1 cells were subjected to SIN-1 at indicated concentration for 4 h and samples were assessed for de novo translation by ribopuromycylation. Actin was used as a loading control. **e** U2OS cells were stressed with 2 mM H_2_O_2_ (1 h), 100 µM SA (1 h), 5 mM SIN-1 (4 h) or left untreated (control). Cap binding assay was performed with m^7^GTP-agarose. **f**, **g** SG-positive cells were quantified by immunofluorescence detecting G3BP1 and TIA-1. **f** Wild type or eIF2α^S51A^ MEFs were treated with 2.5 mM SIN-1 for 4 h. **g** Wild type U2OS were treated with 5 mM SIN-1 for 4 h with or without ISRIB (2 nM). Experiments are represented as mean ± SEM, *p < 0.05, *n* ≥ 3 and profiles are representative of at least three independent experiments

SGs are assembled as a result of translation initiation inhibition. To quantify NO-induced translation repression, we used ribopuromycylation, a method that relies on the ability of puromycin to incorporate and terminate polypeptide chain elongation, to quantify translation. U2OS cells were pulsed with puromycin and its relative incorporation was determined by western blotting with an antibody against puromycin. Both SA and SIN-1 inhibit translation in a dose-dependent manner (Fig. 2a). Intriguingly, SA and SIN-1 produce distinctive changes in the polysome profiles even when the effects on global translation are similar (Fig. 2a, b, compare SA 50 µM and SIN-1 5 mM). In cells treated with SA, heavier polysomes are selectively depleted, whereas SIN-1 depletes polysomes across the profile (Fig. 2b) suggesting that the mechanism of translational repression may be different.

SIN-1 induces phosphorylation of eIF2α in a concentration-dependent manner (Fig. 2c). We therefore sought to determine if phosphorylation of eIF2α is required for SIN-1-induced translation repression and SG formation. In HAP1 cells, SIN-1-induced translational repression is only partially inhibited when the phosphorylation site of eIF2α (by stress- activated kinases at serine residue at the position 51) is mutated to render it non-phosphorylatable (eIF2α^S51A^) (Fig. 2d). This result indicates that SIN-1 predominantly inhibits protein synthesis in a phospho-eIF2α-independent manner. An alternative mechanism of global translation repression centers on the mTOR pathway that targets the eIF4F cap-binding complex. m^7^GTP, the analogue of the cap structure found at the 5′ end of mRNAs, interacts with the cap binding protein eIF4E in association with eIF4A and eIF4G (aka eIF4F), important  to initiate translation. Alternatively, eIF4E can bind 4EBP (eIF4EBP1) to assemble a complex that inhibits translation. m^7^GTP-agarose pulls down both complexes from cell lysates. Whereas the ratio of these complexes is not changed in cells treated with SA, the inhibitory eIF4E:4EBP complex predominates in cells treated with either H_2_O_2_ or SIN-1 (Fig. 2e). Thus, SA inhibits translation predominantly by phosphorylating eIF2α, whereas H_2_O_2_ and SIN-1 inhibit translation predominantly by displacing the eIF4F complex from the m^7^GTP cap.

Mouse embryonic fibroblasts (MEFs) derived from mice expressing mutant eIF2α^S51A^, do not assemble SIN-1 induced granules (Fig. 2f). We also co-treated cells with SIN-1 and the integrated stress response inhibitor (ISRIB), a drug that renders cells unresponsive to the inhibitory effects of eIF2α phosphorylation^14,15^, and found that they no longer assemble SGs (Fig. 2g). Thus, unlike SIN-1-induced translational repression, SIN-1 induced SG assembly is dependent upon phosphorylation of eIF2α.

Lastly, we performed a time course to determine the kinetics of the various signaling events. We found that SIN-1-induced phosphorylation of eIF2α occurs within 1 h, whereas dephosphorylation of 4EBP occurs after 2–3 h (Figure S2). These results suggest that NO-induced inhibition of protein synthesis results from both phosphorylation of eIF2α and displacement of the eIF4F complex as a result of 4EBP dephosphorylation.

### NO induces atypical SGs

**Fig. 3:**
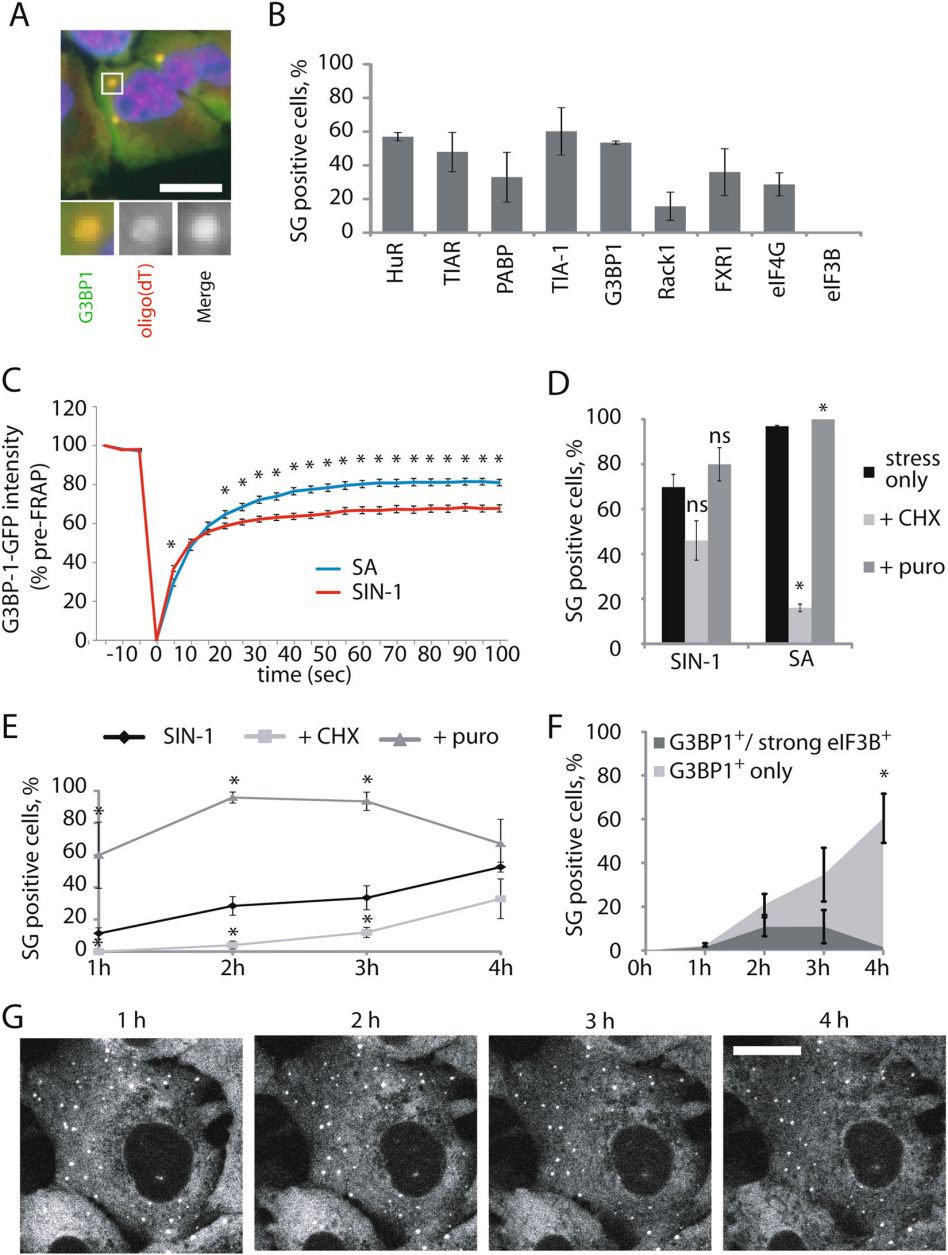
NO induces atypical SGs. **a**–**c** U2OS cells were subjected to 5 mM SIN-1 for 4 h. Scale bar is 10 µm. **a** Representative image of FISH detecting poly(A) RNA and immunofluorescence detecting G3BP1. **b** Quantification of recruitment of typical SG markers to SIN-1 induced SGs. **c** U2OS cells knocked out for endogenous G3BP1 and stably expressing a GFP tagged version of G3BP1 were used. G3BP1-GFP SIN-1-induced SGs were photo-bleached and fluorescence intensity was recorded including 3 images before bleaching, during bleaching as indicated by the intensity drop and after bleaching. Data is normalized to the pre-bleached level, and represents a combination of 3 to 4 experiments with 8-12 SGs per experiment. Cells were treated with SA (100 µg/ml) for 1 h before data collection, or with SIN-1 (5 mM) for 4 h. **d**, **e** Puromycin (20 µg/ml) or cycloheximide (50 µg/ml) were added 30 min before collection. SIN-1 incubation time is as indicated or 4 h. SA was used at 100 µM for 1 h. **f** Cells were collected at indicated times and stained for G3BP1 and eIF3B. At least 3 images were taken by time each point and SG positive cells for each marker were quantified and graphed. **g** U2OS knocked out for endogenous G3BP1 and stably expressing a GFP tagged version of G3BP1 were used. Cells were subjected to SIN-1 5 mM, and pictures were collected at 1, 2, 3, and 4 h of treatment. Only G3BP1-GFP channel was recorded. Experiments are represented as mean ± SEM, *p < 0.05, *n* ≥ 3 and pictures are representative of at least three independent experiments. Scale bar is 10 µm

The composition of SGs varies with the inducing stress and compositional differences have been suggested to influence SG function(s)^2,16^. Therefore, we characterized the contents of SIN-1-induced SGs by immunofluorescence microscopy at 4 h of SIN-1 treatment. SIN-1-induced SGs contain poly(A) RNAs (oligo(dT) labeling), TIA-1, TIAR, G3BP1, HuR, PABP, FXR1, eIF4G, and RACK1 but exclude eIF3B (Fig. 3a, b). As eIF3B is a prominent component of SA-induced SGs in U2OS cells^7^, its absence is a distinguishing feature of SIN-1-induced SGs. eIF3D, eIF3G and eIF3J subunits are also excluded from SIN-1-induced SGs (Figure S3) suggesting that the entire eIF3 complex is absent.

SGs are dynamic structures whose components constantly exchange with the cytoplasm^17^. To probe the dynamic nature of SIN-1 induced SGs, we performed fluorescence recovery after photobleaching (FRAP) in cells knocked out for endogenous G3BP1 and stably expressing a GFP-tagged version of G3BP1. This approach avoids artefacts induced by overexpression of G3BP1^18^. Fluorescence recovery was modestly, but significantly, reduced in SIN--1 verses SA-induced SGs (Fig. 3c), suggesting that SIN-1-induced SGs have a greater immobile fraction than canonical SA-induced SGs.

To further interrogate the dynamic nature of SIN-1-induced SGs, we used two drugs that have opposite effects on SG assembly: cycloheximide (CHX), which “freezes” ribosomes on mRNAs and disassembles SGs, and puromycin (puro), which promotes polysome collapse and enhances SG assembly. Whereas CHX strongly inhibits the formation of SA-induced SGs, it only modestly inhibits the assembly of SIN-1 induced SGs at the 4 h time point (Fig. 3d). Under these conditions, puromycin significantly enhances the formation of SA-induced SGs, but does not significantly enhance the assembly of SIN-1-induced SGs (Fig. 3d). A time course analysis reveals that SGs assembled at early times are significantly more dynamic than those assembled at later times (Fig. 3e). Using live cell imaging, we followed after a SG-positive cell between 1 and 4 h after the addition of SIN-1. We noticed that SIN-1-induced SGs are not as mobile as SA-induced SGs. Moreover, SIN-1-induced SGs do not undergo fusion or fission events and they have limited (until 3 h) or no (after 3 h) movement in the cytoplasm (Fig. 3g). This phenomenon correlates with the temporal loss of eIF3B: whereas eIF3B is a prominent component of SGs analyzed at early time points, it is largely absent from SGs analyzed at later time points (Fig. 3f). Taken together our data suggest that over time SIN-1-induced SGs that lose eIF3B become less dynamics and less mobile.

### NO-induced type II SGs correlate with decreased cell viability

**Fig. 4:**
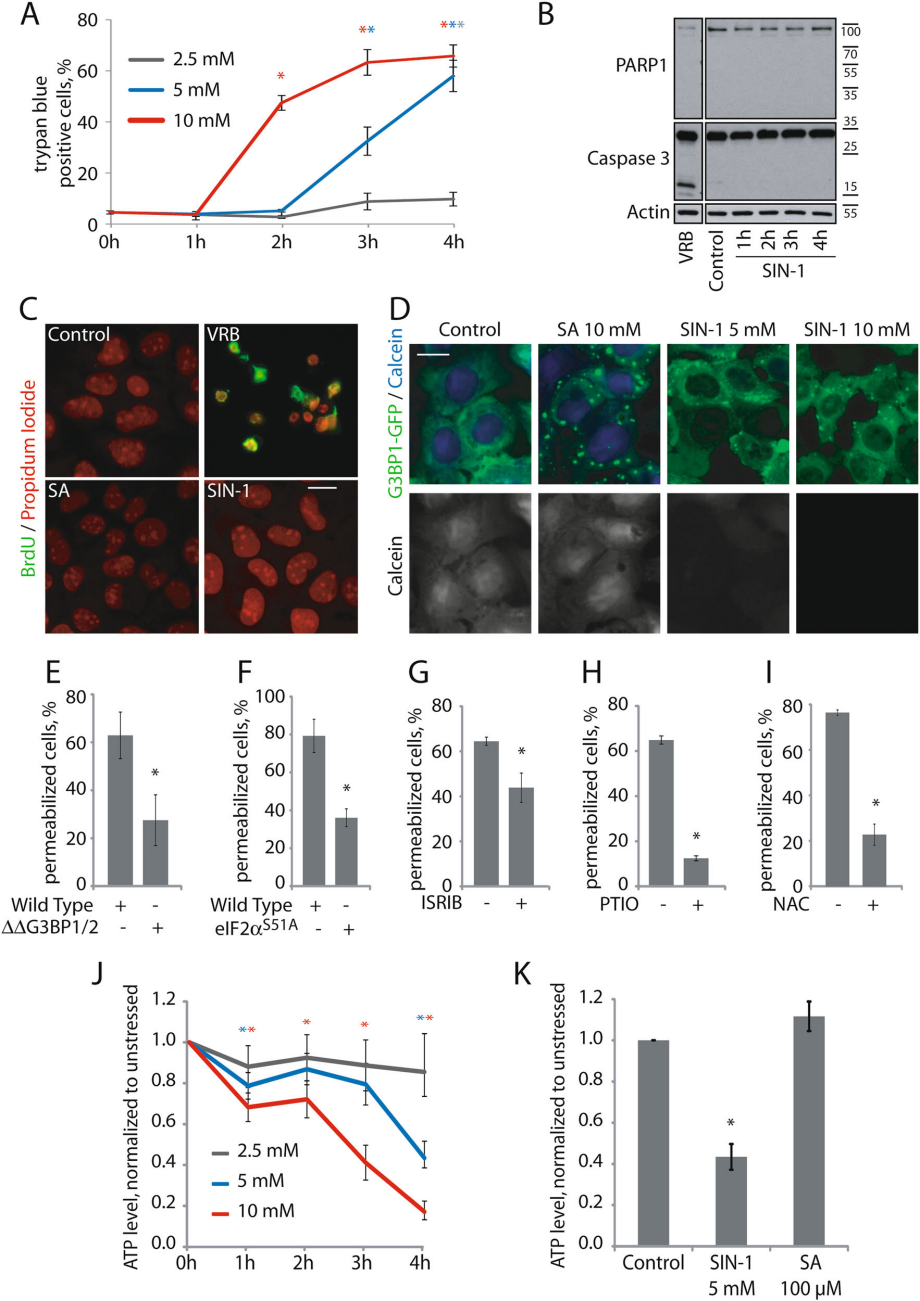
Formation of NO-induced atypical SGs correlates with increased cell death. **a**–**d** U2OS were treated with 5 mM SIN-1 for the indicated times. **a** Cell permeabilization was assessed by manual trypan blue exclusion cell counting. **b**, **c** U2OS cells were stressed with 5 mM SIN-1 for 4 h. Vinorelbin (VRB) was used as a positive control for Caspase 3 activation. SA was used at 100 µM for 1 h. **b** Cells were lysed every hour and analyzed by western blot. Key apoptosis proteins were investigated. Actin was used as a loading control. **c** Representative images demonstrating BrdU and propidium iodide incorporation. **d** U2OS ΔG3BP1 overexpressing G3BP1-GFP cells were incubated with 5 µM Calcein blue AM for 5 min. **e**–**i** Cell permeabilization was assessed by trypan blue. **e** Wild type and ΔΔG3BP1/2 U2OS cells or **f** wild type or eIF2α^S51A^ MEFs were treated with SIN-1 5 mM (U2OS) or 2.5 mM (MEFs) for 4 h. **g**–**i** U2OS cells were treated with SIN-1 (5 mM) for 4 h with or without **g** ISRIB (2 nM), **h** PTIO (5 mM), or **i-** NAC (5 mM). **j**, **k** U2OS cells were stressed with SIN-1 at indicated concentrations for the indicated times and with 100 µM SA 1 h. ATP level was measured using Celltiter glo and plotted. Experiments are represented as mean ± SEM, *p < 0.05, *n* ≥ 3

eIF3B-deficient SGs have been linked to increased cell death. Like SIN-1, supranutritional levels of selenite generate ROS and trigger the formation of eIF3B-deficient SGs^5^. SIN-1 induces a dose-dependent induction of trypan blue uptake (Fig. 4a). SIN-1- induced trypan permeabilization is not accompanied by the cleavage of Caspase-3 or PARP1 which accompanies vinorelbine (VRB)-induced apoptosis (Fig. 4b)^19^. Similarly, VRB but not SA or SIN-1 induces nuclear condensation and BrdU labeling, a characteristic of apoptotic cell death (Fig. 4c)^19^. We also used Calcein blue AM, a membrane-permeable live-cell labeling dye, to assess membrane integrity. Upon entering the cell, intracellular esterases cleave the acetoxymethyl ester group of calcein leading to viewable labeling under UV light. Cells with compromised cell membranes do not retain calcein. Untreated cells and SA-treated cells retain calcein, denoting that they all have intact plasma membranes. In contrast, SIN-1-treated cells no longer retain the dye, thus indicating a loss of plasma cell membrane integrity (Fig. 4d). We also demonstrate that SNAP increases the trypan blue cell permeability (Figure S1, light bar). These results suggest that NO induces non-apoptotic cell permeabilization.

To determine whether NO-induced membrane permeabilization is influenced by SG assembly, we analyzed mutant cell lines deficient for SG assembly. In both U2OS^ΔΔG3BP1/2^ and MEF (eIF2α^S51A^) cells, NO-induced membrane permeabilization is significantly reduced compared to WT controls (Fig. 4e, f). Moreover, ISRIB, PTIO and NAC, all of which inhibit SG assembly (Figs. 1g–h and 2g respectively), also inhibit NO-induced membrane permeabilization when used in combination with SIN-1 as compared to SIN-1 alone (Fig. 4g–i), indicating that phosphorylation of eIF2α and ROS contribute to this functional response. Together these data suggest that SIN-1-induced cell permeabilization is due to NO, and it is increased when SGs are formed.

Non-apoptotic membrane permeabilization could be a feature of necrotic cell death, which is typically triggered by decreased cellular ATP levels^20^. Indeed, SIN-1 decreases ATP levels in a dose- and time-dependent manner (Fig. 4j). In contrast, SA does not reduce cellular ATP levels (Fig. 4k). Thus, SIN-1-induced assembly of type-II SGs, but not SA-induced assembly of type-I SGs is associated with depletion of ATP and membrane permeabilization. Thus, loss of membrane permeability (trypan blue and calcein staining) and decrease of cellular ATP is linked to the assembly of type II SGs.

### NO disrupts mitochondria morphology and function

**Fig. 5:**
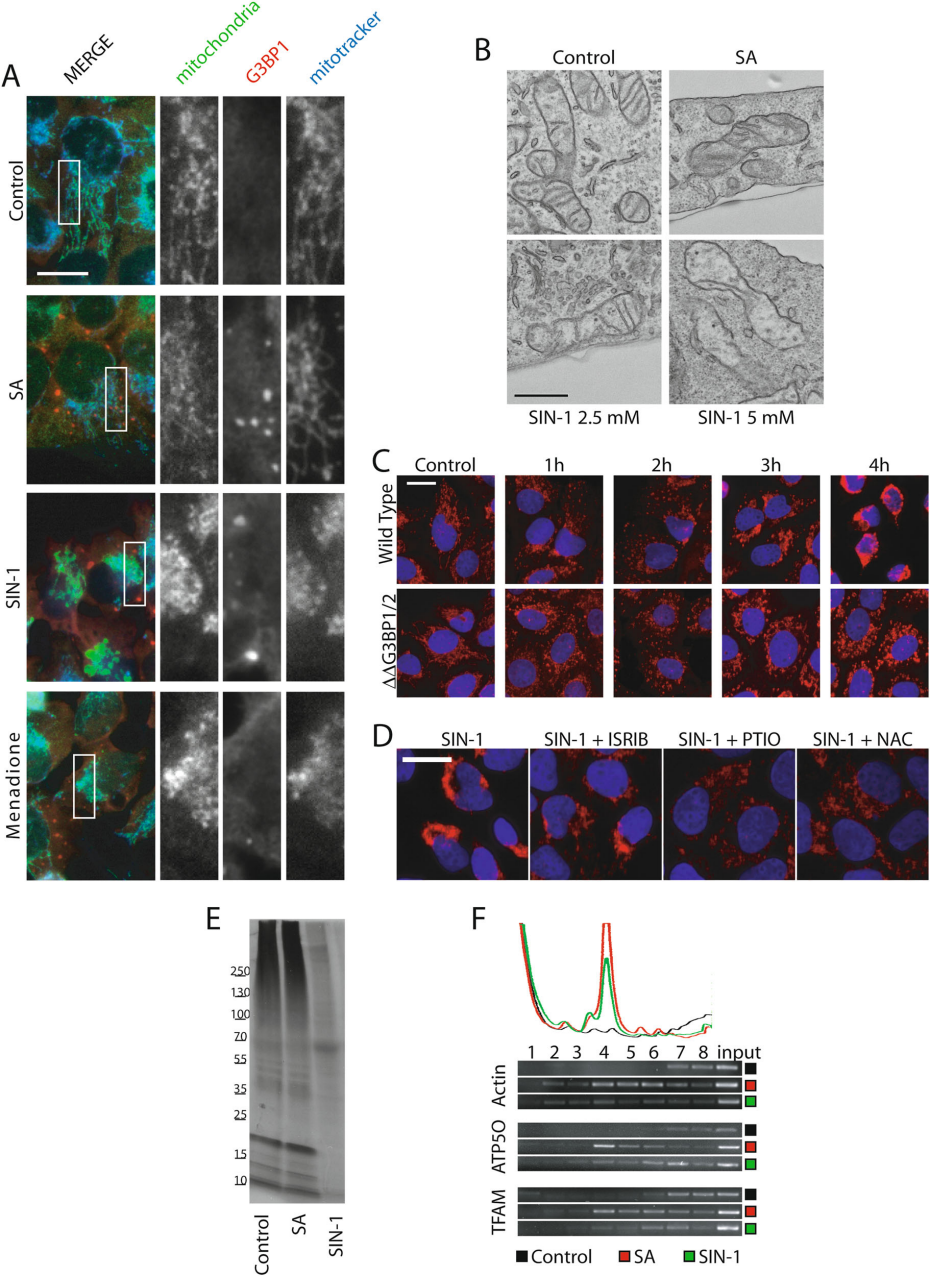
NO induces mitochondrial damage. **a**, **b** U2OS cells were treated with SIN-1 (5 mM) or at indicated concentrations for 4 h, SA (100 µM) for 1 h, menadione (100 µM) for 1 h or left untreated (Control). **a** To follow mitochondria integrity, Mitotracker was added to the media 15 min before collection. In addition, cells were stained with a mitochondria-specific human auto-antibody. Enlarged sections of the white boxes are presented on the right panels. Scale bar is 10 µm. **b** Electron microscopy representative images. Scale bar is 500 nm . **c** U2OS wild type or U2OS^ΔΔG3BP1/2^ cells were treated with SIN-1 and stained with Hoechst dye (blue) and mitochondria-specific antibody (red) at indicated times. **d** U2OS cells were treated with SIN-1 (5 mM) for 4 h with or without ISRIB (2 nM), PTIO (5 mM), or NAC (5 mM). Cells were labeled using Hoechst (blue) and anti-mitochondria antibody (red). **e** To assess mitochondrial translation cells were incubated with labeling media without methionine for 30 min, then emetine was added 5 min. Then cells were incubated in the labeling media containing emetine and [^35^S]-Met for 1 h. Translation was assayed by autoradiography. Wild type cells were stressed with SIN-1  (5 mM) for 4 h, SA (100 µM) for 1 h or left unstressed. **f** Cells were treated with SIN-1 (5 mM) for 4 h, SA (100 µM) for 1 h or were not treated. Samples were collected and loaded onto a 10–50% continuous sucrose gradient. Polysome profiles were assessed (upper panel) and fractions were collected to purify associated RNAs and to perform semi-quantitative PCR (lower panel). Experiments are *n* ≥ 3

The finding that NO induces ROS production (Fig. 1e, f) and decreases cellular ATP (Fig. 4j, k) led us to examine the effect of NO on mitochondria, the major source of cellular ROS and ATP. In untreated and SA-treated cells, we observed perfect co-localization between anti-mitochondrial antibody staining and mitotracker, a marker of intact mitochondrial function, at long interconnected mitochondria (Fig. 5a). Both SIN-1 and the mitochondrial poison menadione (used as a control for mitochondrial damage) severely disrupt mitochondrial morphology and function (Fig. 5a). Like SIN-1, menadione induces the formation of type-II SGs (Fig. 5a, bottom panel and S4A), and loss of calcein labeling (Figure S4B). We verified mitochondria disruption following SIN-1 exposure in U2OS cells using electron microscopy, which revealed mitochondrial swelling and disruption of cristae that increased with SIN-1 concentration (Fig. 5b). NO-induced mitochondrial damage is progressive over a 4 h time course, culminating in a severe mitochondrial aggregation (Fig. 5c, upper right panel). In contrast, U2OS^ΔΔG3BP1/2^ cells that are unable to assemble SGs exhibit mitochondrial fragmentation, but not aggregation (Fig. 5c, lower right panel). Those cells also exhibit reduced ROS production compared to WT controls (Figure S5).

We also observed that compounds that inhibit SIN-1-induced SG assembly (ISRIB, PTIO and NAC; Figs. 1g–h and 2g respectively), and membrane permeabilization (Fig. 4g–i), also inhibit mitochondrial aggregation (Fig. 5d). The effects of PTIO and NAC are more potent than ISRIB (Fig. 5d), consistent with their effects on membrane permeabilization (Fig. 4g–i).

Stress-induced re-programming of protein translation plays a major role in protecting cells from deleterious effects of stress^21–24^. Given the central role of mitochondria in NO-induced cell death, we determined whether NO also affects mitochondrial protein synthesis. We first used immunoblotting to confirm that the expression of nuclear and mitochondrial encoded mitochondrial proteins is similar during a 4 h exposure to SIN-1 (Figure S6). The mitochondrial genome encodes 18 genes that are transcribed and translated entirely within mitochondria^25^. Mitochondrial protein synthesis can be quantified using ^35^S-pulse labeling in conjunction with emetine, a drug that inhibits the cytosolic 80S ribosome-mediated protein synthesis but does not inhibit mitochondrial 55S ribosome-mediated translation^26^. Using this method, we observed robust mitochondrial translation under control conditions and in cells treated with SA (Fig. 5e). In contrast, a 4 h exposure to SIN-1 strongly and selectively inhibits mitochondrial protein synthesis (Fig. 5e).

Since most mitochondrial proteins are nuclear encoded, we also investigated the translation of nuclear encoded mitochondrial proteins. Stressed U2OS cells were processed for polysome gradient analysis followed by semi-quantitative RT-PCR. Similar to *Actin* mRNA, *ATP50* and *TFAM* mRNAs (both nuclear encoded mitochondrial proteins) are concentrated in the heaviest polysomes under control conditions. These transcripts shift to the monosome fractions in cells treated with SA or SIN-1 (Fig. 5f), although the distribution of *TFAM* mRNAs toward lighter polysomal fractions is not as dramatic as for *Actin* or *ATP5O* mRNA: while majority of the tested mRNAs being found in the 4th fraction, the distribution of *TFAM* mRNA was throughout the gradient, suggesting weak translation. In response to 5 mM SIN-1, the *ATP50* and *TFAM* mRNA profiles shift towards the lighter fractions but not to the same extent as with SA suggesting a reduced but not entirely repressed translation of those mRNAs (Fig. 5f). Taken together we show that SIN-1 induces mitochondrial damages associated with the inability to perform mitochondrial translation.

## Discussion

NO has multiple effects on cell physiology^27–29^, and NO dysregulation has been implicated in neurodegenerative disease, allograft rejection, and cancer^30–32^. Some controversy exists regarding the molecular mechanism of NO action as it has been reported to have both pro-survival and pro-death properties^33,34^. These functional effects have been attributed to the promotion of mitochondria biogenesis or the inhibition of mitochondrial respiration, respectively^35,36^. Our results show that NO sequentially promotes the assembly of pro-survival, type-I SGs, and pro-death, type-II SGs that may contribute to the opposite functions of this chemical mediator.

### NO promotes the sequential formation of type-I and type-II SGs

SG assembly was initially correlated with enhanced survival of cells subject to environmental stress^37–41^. SA is a standard for induction of pro-survival type-I SGs, as inhibiting their formation correlates with increased cell death^2,37^. We find that SGs triggered by NO have slow induction kinetics, and phosphorylation of eIF2α is required for their formation (Figs. 1b and 2f). At earlier time points (1–2 h), a minority of cells have SGs that are compositionally similar to SA-induced SGs and are highly dynamic (Fig. 3e–g). Like SA, NO induces inhibition of global protein synthesis and phosphorylation of eIF2α (Fig. 2a–c). Unlike SA, NO also displaces eIF4F from m^7^GTP, a probable consequence of hypophosphorylation of 4EBP, which also contributes to the inhibition of global protein synthesis (Fig. 2e, S2). Over time, NO-induced SGs loose eIF3 and become less dynamic (Fig. 3e–g). During this transition, cells become permeable to trypan blue and lose calcein labeling (Fig. 4a, d), changes associated with plasma membrane permeabilization and cell death. Pharmacologic or genetic inhibition of SG assembly significantly inhibits membrane permeabilization (Fig. 4e–i), linking the transition to type-II SGs with decrease in intracellular ATP levels and loss of viability (Fig. 4j). These data suggest that NO induces the assembly of two different classes of SGs that differ in composition and function: type-I SGs (e.g., those formed in response to SA) include the eIF3 subunits and promote cell survival^2,37^, and type-II SGs (e.g., those described here) lacking eIF3 subunits and linked to mitochondrial damage and decreased survival. We also show evidences for conversion of the type-I granules into the type-II granules (Fig. 3f, g).

### NO-induced mitochondrial damage is linked to ROS production, ATP depletion, SG assembly, and membrane permeabilization

The finding that NO induces ROS production (Fig. 1e) and ATP depletion (Fig. 4j) suggests that the mitochondria may be a proximal target of NO toxicity. Indeed, NO markedly disrupts mitochondrial structure and function (Fig. 5a, b). In contrast, SA that fails to induce ROS production or ATP depletion has no effect on mitochondrial morphology or function (Figs. 1e and 4k). In response to NO, mitochondria loose internal cristae concurrent with mitochondrial network disruption (Fig. 5a, b). The reticular mitochondrial network is progressively converted into aggregates beginning at 3 h (Fig. 5c), coincident with accelerating ATP depletion (Fig. 4j) and cell membrane permeabilization (Fig. 4a, d).

### Mitochondrial protein translation is linked to cell survival under ROS exposure

Protein translation is an energy consuming process. In stressed cells, global translation is reduced, allowing cells to conserve energy for the repair of stress-induced damage^21–24^. It is known that NO induces translational repression^3^. Here, we show that this is due to early phosphorylation of eIF2α (Figure 2), and subsequent dephosphorylation of 4EBP (Figure 2), modifications that deplete the eIF2α-GTP-tRNAi^Met^ ternary complex or displace the eIF4F complex from m^7^G cap structures, respectively. Whereas phosphorylation of eIF2α is required for the formation of NO-induced SGs, mTOR inactivation appears to be selectively needed for the transition to type-II SGs.

Most mitochondrial proteins are nuclear encoded and the translation of key proteins involved in mitochondrial biogenesis and function are regulated via the mTOR pathway^42^. In addition, eIF3 selectively activates the translation of electron transport chain (ETC) proteins required for the generation of ATP^43^. Although sequestration and subsequent release of eIF3 from NO-induced SGs might differentially affect the translation of mitochondrial proteins required for ATP production, we did not observe changes in the expression of ETC proteins over the course of these experiments (Figures 5 and S5).

### Implication for human diseases

This study provides evidence that NO can induce the assembly of two types of SGs. Type-I SGs (e.g., formed in response to SA) include eIF3 complex and assembled early in response to phosphorylation of eIF2α to promote cell survival. Type-II SGs are assembled later, probably in response to the hypophosphorylation of 4EBP. NO-induced type-II SGs are linked to mitochondrial damage, production of ROS, depletion of ATP, and plasma membrane permeabilization.

These findings suggest that type-II SGs act downstream of mitochondrial damage to promote ATP depletion, plasma membrane permeabilization and cell death. Interestingly, type-I and type-II SGs have been reported to modulate toxicity of selected chemotherapeutic agents^5,19,44,45^. Bortezomib induces the formation of type-I SGs that enhance cell survival and limit effectiveness of the drug. In contrast, selenite induces the formation of type-II SGs that enhance cell death^5,44^. The difference in cell outcome after formation of one or the other type of granule could be potentiated in order to develop better or predictive therapy.

## Electronic supplementary material

Supplementary Text

Supplementary Figure 1

Supplementary Figure 2

Supplementary Figure 3

Supplementary Figure 4

Supplementary Figure 5

Supplementary Figure 6
