# Supplementary material for: Nitric oxide triggers the assembly of “type II” stress granules linked to decreased cell viability
Source: Cell Death Dis. 2018 Nov 13;9(11):1129. doi: 10.1038/s41419-018-1173-x (PMC6234215; doi:10.1038/s41419-018-1173-x)
Supplement: Supplementary file 1 — Supplementary Text [file 41419_2018_1173_MOESM1_ESM.docx]

**Figure S1**

U2OS cells were treated with 5 mM SNAP for 7 h. Cells were assessed as SG positive by G3BP1 and plotted into the graph (dark bar). Cell permeabilization was assessed by trypan blue (light bar). Experiments are represented as mean ± SEM, n≥2.

**Figure S2**

U2OS cells were treated with 5 mM SIN-1 and collected every hour, lysed and analyzed by western blot with indicated antibodies.

Data are representative of ≥3 independent experiments.

**Figure S3**

U2OS were treated or not with SA, 100 µM, 1 h, or SIN-1, 5 mM at indicated time. Cells were stained using indicated antibodies and shown as merged (MERGE) or as individual factor-specific channel.

Images are representative of 3 independent experiments. Scale bars are 10 µm

**Figure S4**

U2OS were treated or not with SA, 100 µM, 1 h, or Menadione 100 µM, 1 h.

**A-** Cells were labelled using eIF3b and G3BP1 antibody as well as Hoechst.

**B-** Cells were incubated with 5 µM Calcein blue AM for 5 min.

Picture are representative of n=3 independent experiments. Scale bars are 10 µm

**Figure S5**

30 min before collection, U2OS wild type or ΔΔG3BP1/2 were incubated with 2.5 µM CellRox for the remaining time. Images are taken directly after fixation. CellRox intensity is expressed relative to the unstressed control intensity.

**Figure S6**

U2OS cells were collected as indicated, lysed and analyzed by western blot with indicated antibodies. Experiments are represented as mean ± SEM, n≥3.
