# Supplementary figures and images for: Nitric oxide triggers the assembly of “type II” stress granules linked to decreased cell viability
Source: Cell Death Dis. 2018 Nov 13;9(11):1129. doi: 10.1038/s41419-018-1173-x (PMC6234215; doi:10.1038/s41419-018-1173-x)

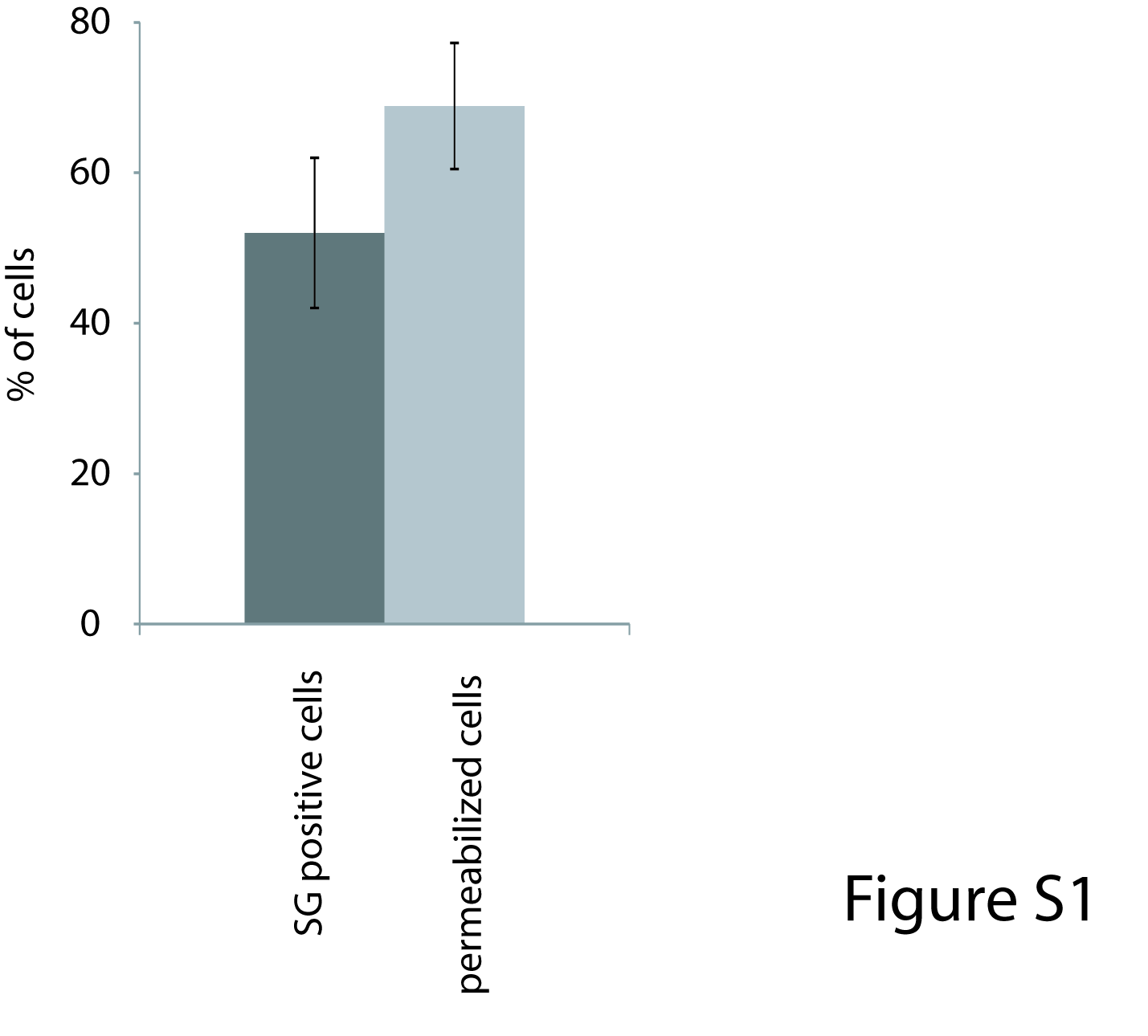

Supplement: Supplementary file 2 — Supplementary Figure 1 [file 41419_2018_1173_MOESM2_ESM.tif]

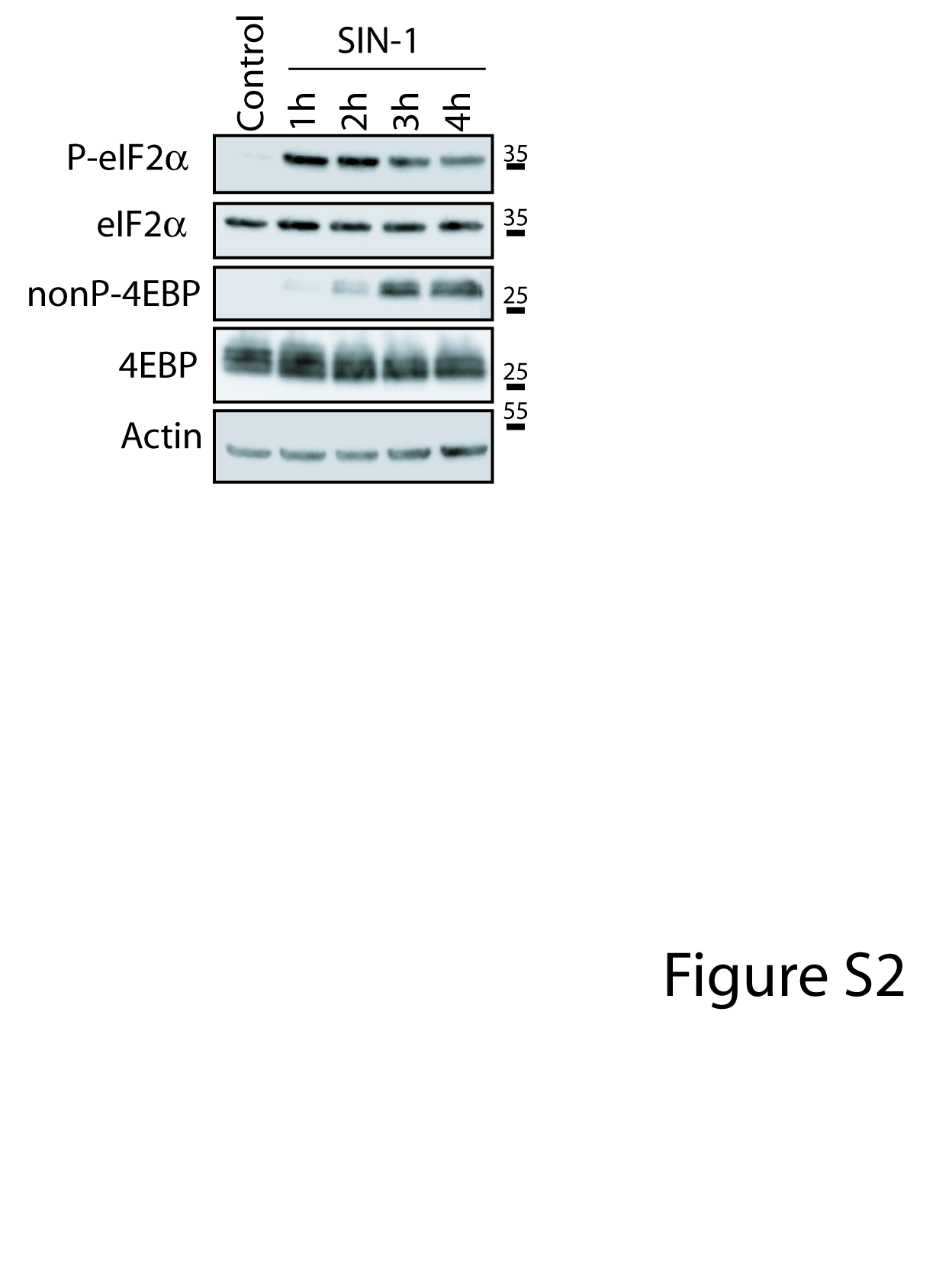

Supplement: Supplementary file 3 — Supplementary Figure 2 [file 41419_2018_1173_MOESM3_ESM.tif]

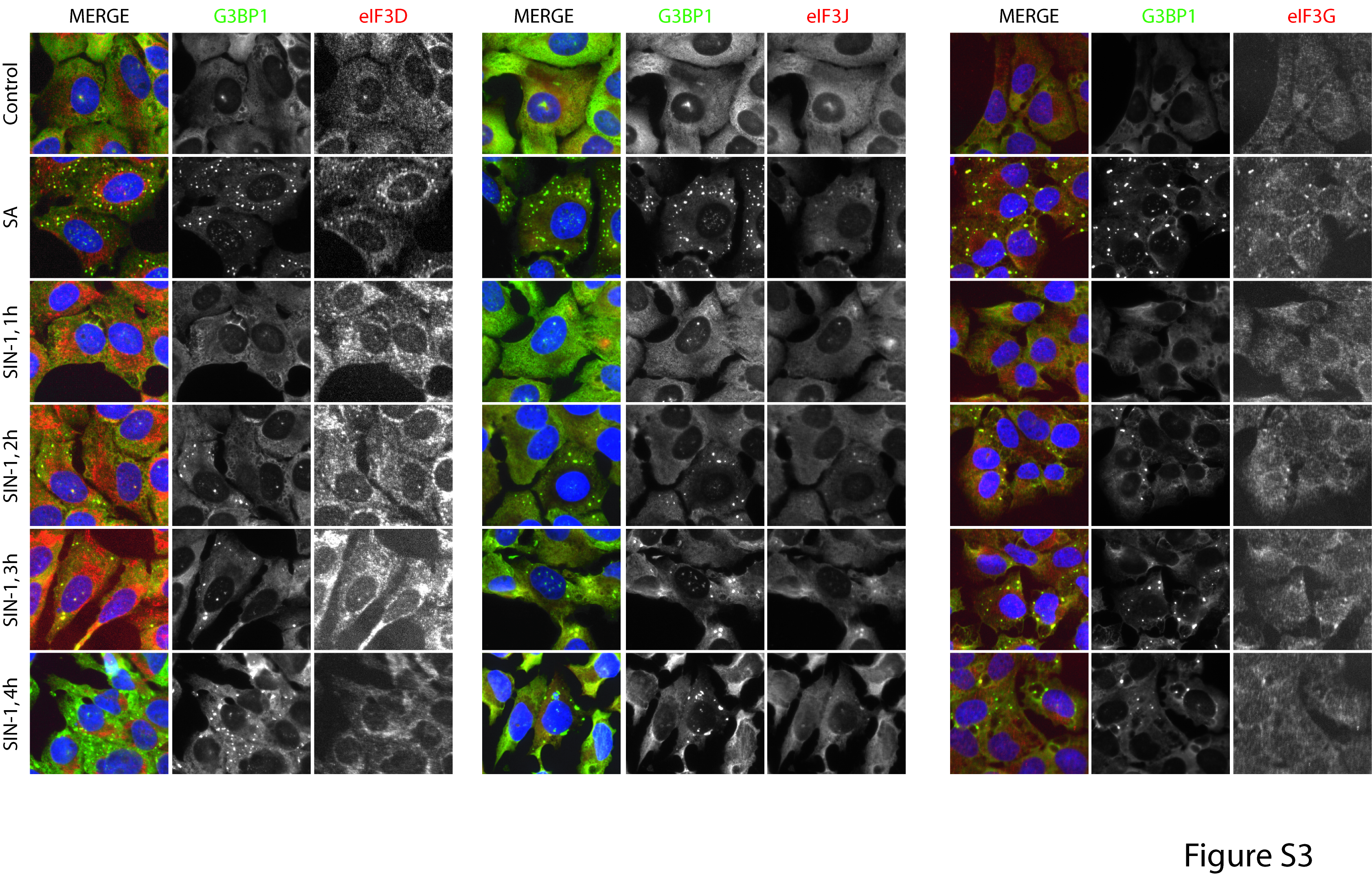

Supplement: Supplementary file 4 — Supplementary Figure 3 [file 41419_2018_1173_MOESM4_ESM.tif]

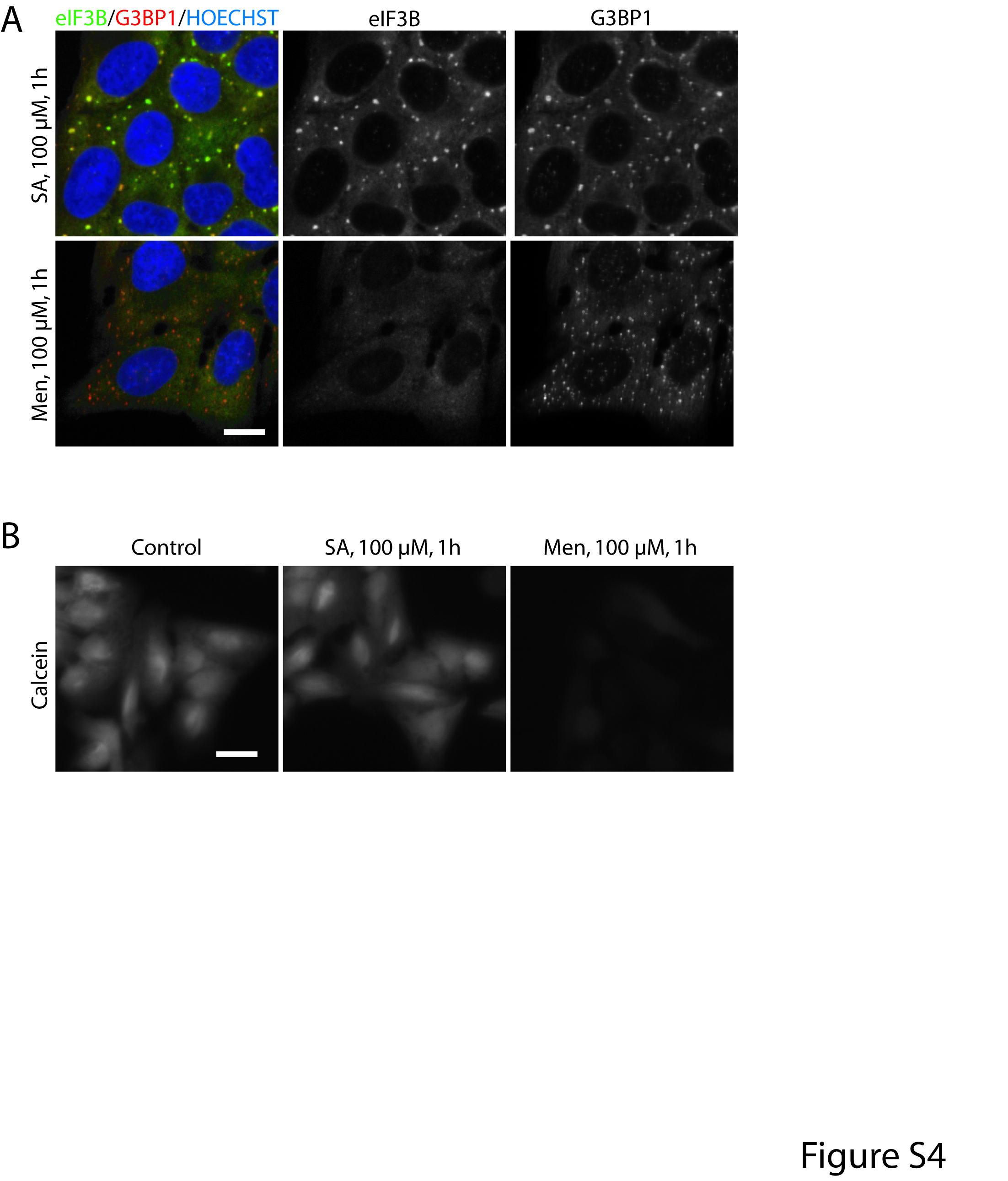

Supplement: Supplementary file 5 — Supplementary Figure 4 [file 41419_2018_1173_MOESM5_ESM.tif]

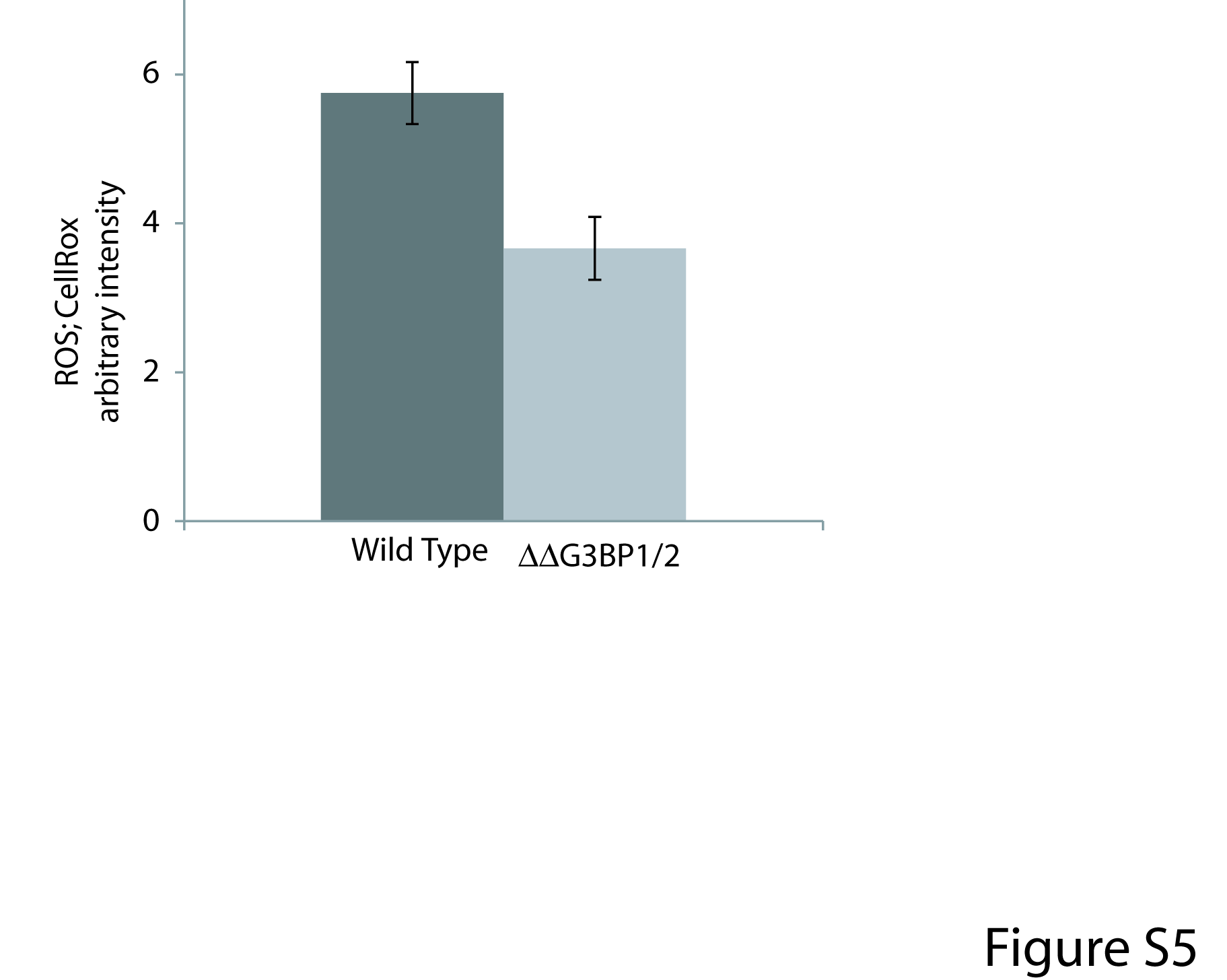

Supplement: Supplementary file 6 — Supplementary Figure 5 [file 41419_2018_1173_MOESM6_ESM.tif]

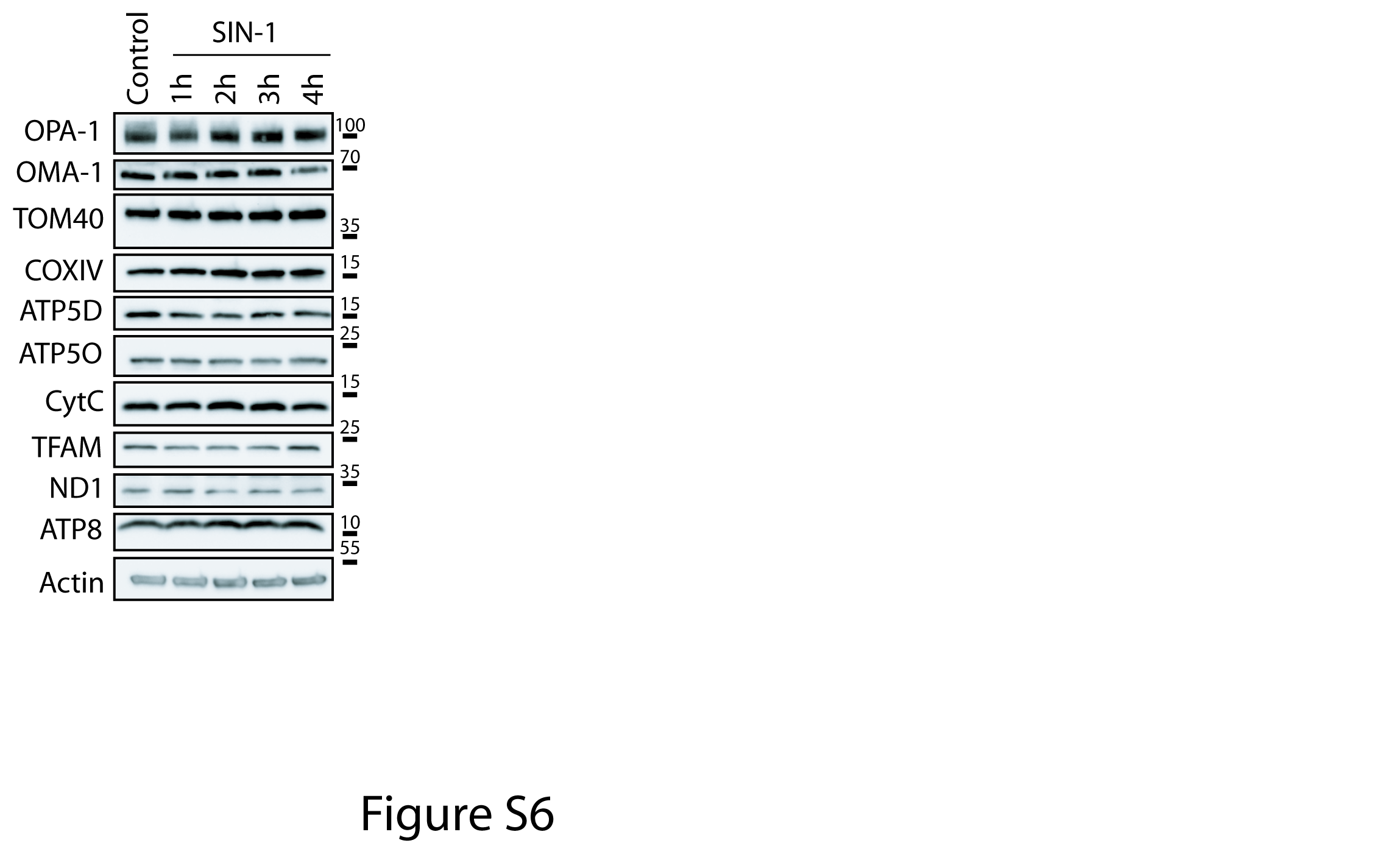

Supplement: Supplementary file 7 — Supplementary Figure 6 [file 41419_2018_1173_MOESM7_ESM.tif]
